# Primary Non-Hodgkin’s Lymphoma of the Spine: A Case Report and Literature Review

**DOI:** 10.14740/wjon947w

**Published:** 2015-10-26

**Authors:** Elias Moussaly, Bassel Nazha, Mazen Zaarour, Jean Paul Atallah

**Affiliations:** a Department of Internal Medicine, Staten Island University Hospital, North Shore - LIJ Health System, 475 Seaview Avenue, Staten Island, New York 10305, USA; b Division of Hematology and Oncology, Department of Internal Medicine, Staten Island University Hospital, North Shore - LIJ Health System, 475 Seaview Avenue, Staten Island, New York 10305, USA

**Keywords:** Non-Hodgkin’s lymphoma, Extranodal lymphoma, Diffuse large B cell lymphoma, Spinal tumor

## Abstract

Primary non-Hodgkin’s lymphoma (NHL) of the spine is a rare form of extranodal lymphoma. This entity constitutes a diagnostic challenge due to its mimicking of other spinal diseases and the difficulty in establishing a tissue diagnosis. In fact, core biopsy can be inconclusive, oftentimes requiring surgical biopsy. Definitive evidence is lacking regarding the treatment of choice. As a result, the prognosis remains unfavorable. We present the case of an adult female who presented with back pain and was found to have a spinal NHL. We also review the literature regarding this rare occurrence.

## Introduction

Primary spinal lymphoma is a rare form of extranodal lymphoma. It is thought to arise from the paraspinal lymphoid tissue and subsequently invade the spinal cord. The diagnosis of this entity is challenging due to its atypical clinical presentation and the difficulty in establishing a conclusive tissue diagnosis with core biopsy. The treatment of choice has yet to be defined, and the role of surgery remains controversial. Therefore, the prognosis of this entity remains unfavorable. We present the case of an adult female who presented with back pain and was found to have a spinal lymphoma. We also review the literature regarding this rare occurrence.

## Case Report

A 69-year-old Caucasian female with a past medical history of hypertension, rheumatoid arthritis, Sjogren syndrome, and esophagitis presented to our emergency department for evaluation of numbness in her arms and legs. Over the last 6 weeks, she has been experiencing intermittent chest tightness and mid-back pain. Twenty-four hours prior to presentation, she developed constant moderate to severe bilateral paresthesia in her upper and lower extremities. She denied fever, night sweats, shortness of breath, palpitations, recent weight loss or weakness in her extremities.

Six weeks prior to presentation to our facility, the patient had an unrevealing workup for cardiac etiologies of her chest pain at a neighboring hospital. A chest computed tomography (CT) had shown spinal sclerosis from T4 to T7, which was thought to be the etiology of her back pain. A course of non-steroidal anti-inflammatory drug provided partial relief. One month later, she noted worsening of her symptoms. A thoracic magnetic resonance imaging (MRI) with contrast had shown a 4.5 × 1.6 cm fusiform mass in the epidural space from T5 to T7, along with large bilateral paraspinal masses extending from T4 to T9 with invasion into the neural foramina. Two CT-guided biopsies of the spine had been attempted but did not yield enough tissue, and were therefore inconclusive.

The patient’s surgical history included an appendectomy, bilateral total knee replacements, a hysterectomy for uterine fibroids and a breast lumpectomy in the remote past for breast cancer from which she is in remission. The patient has a documented allergy to penicillin, codeine and sulfa drugs. She stopped smoking 20 years ago and did not abuse alcohol or recreational drugs. She was independent in her activities of daily living. The patient’s medication consisted of alendronate, amlodipine, omeprazole, hydrochlorothiazide, vitamin D, methotrexate, pregabalin, calcium, pyridoxine, and vitamin B12. She was previously on adalimumab for her rheumatoid arthritis, which was stopped since the differential diagnosis of her suspicious spinal mass included extrapulmonary tuberculosis.

On physical examination, the patient appeared uncomfortable due to her back pain. She had mildly decreased breath sounds on auscultation with regular heart rate and normal cardiac sounds. Her abdomen was soft and non-tender. There were no palpable cervical or axillary lymph nodes. Neurologic exam was within normal limits, with preserved motor functions, normal reflexes and no noted sensory deficits.

**Figure 1:**
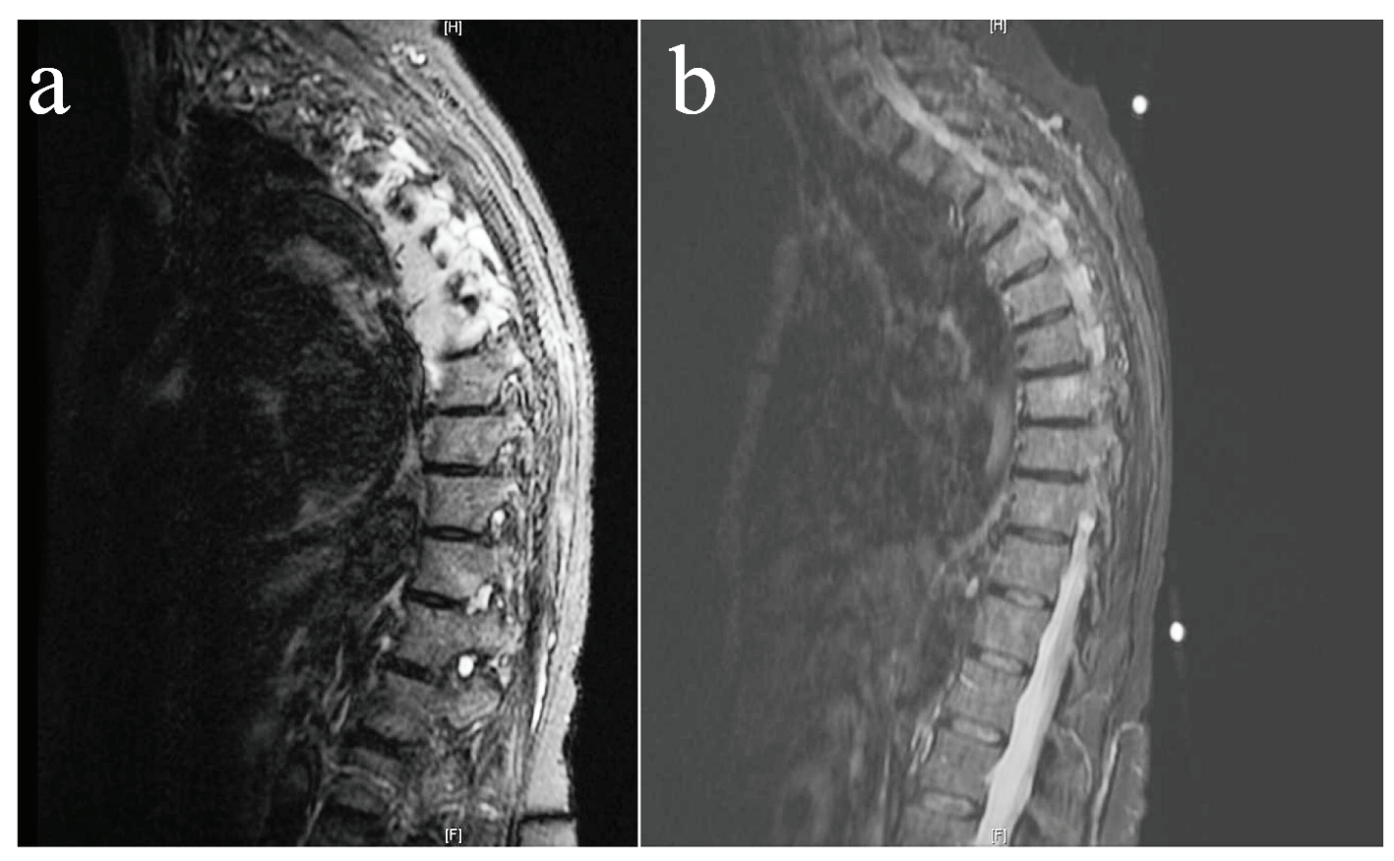
Pretreatment (a) and post-treatment (b) T2-weighted MRI showing a near complete resolution of the paraspinal metastatic disease and epidural soft tissue extension, with no evidence of cord compression after three cycles of R-CHOP and intra-thecal methotrexate.

At our hospital, since she was complaining of chest pain, a CT of the chest with contrast, intended to rule out pulmonary embolism, showed bilateral pleural effusions and an increased soft tissue density in the bilateral paraspinal compartment ranging from T4 to T8, which was suspicious for a malignant process. No endobronchial obstruction or pulmonary mass were found. An MRI of the thoracic spine with contrast subsequently revealed multiple foci of bone marrow signal abnormalities suspicious for malignancy, and infiltration of the T4 to T6 vertebral bodies. Extensions into the neural foramens, spinal canal, adjacent ribs, epidural and paraspinal soft tissue were also noted on multiple levels. Additional osseous lesions were found at T7, T8, and T11. Cord compression was pronounced at the level of T4-T5. The tumor had overall progressed and increased in size compared to the prior MRI study done at another facility (Fig. 1a).

The patient was diagnosed as having cord compression syndrome and promptly received high-dose steroids and pain control medications, after consultation with neurosurgery. She noted that these conservative measures improved her paresthesia. Given a high suspicious of a malignant process, her oncologist performed an extensive workup. An MRI of the brain did not show any intraparenchymal lesions. A CT scan of the abdomen and pelvis revealed no enlarged lymph nodes, and was otherwise normal. A bone marrow biopsy was normocellular with trilineage hematopoesis. Significant blood tests results are shown in Table 1. Further tests for hepatitis B, hepatitis C and HIV were all negative. Electrolytes, kidney function tests and liver function tests were unremarkable.

**Table 1:** Pertinent Laboratory Results on Admission

WBC	13,750/mm^3^
Hemoglobin	12.2 g/dL
Platelet	203,000/mm^3^
ESR	30 mm/h
Phosphorus	3.6 mg/dL
Uric acid	5.8 mg/dL
Alpha fetoprotein	2.1 ng/mL
Ca 125	110 U/mL (high)
Ca 15-3	27.7 U/mL
Ca 19-9	22.2 U/mL
Ca 27-29	27.7 U/mL
Carcinoembryonic antigen	1.8 ng/mL
LDH	268 U/L (high)
Calcium	9.2 mg/dL

In light of her neurological symptoms due to the cord compression and since multiple CT-guided biopsies did not yield a diagnosis, the patient underwent neurosurgical decompressive laminectomies from T4 to T8, with resection of the epidural spinal cord tumors. Significant neurological invasion limited the extent of tumor resection. Analysis of the pathological sections showed a diffuse infiltrate of lymphoid cells consistent mainly of small lymphocytes with significant number of scattered large lymphoid cells with irregular often prominent nucleoli, vesicular chromatin and abundant clear cytoplasm. Most cells were positive for the B cell antigens, CD-20 and PAX-5. The atypical large cells were strongly positive for the B cell antigen CD-20, the germinal cell antigen BCL-6 and the post germinal B-cell antigen MUM-1. BCL-2 and BCL-1 (cyclin D1) were negative as well as CD-10, CD-15, CD-21, CD-23, CD-138 and CD-30. The P-53 regulatory protein was rarely expressed. No Reed Sternberg cells were found. *In situ* hybridization showed a mixed population of kappa and lambda light chain positive plasma cells. The overall pattern was consistent with a diffuse large B cell lymphoma of non-germinal center cell of origin due to MUM-1 positivity and CD-10 negativity. The CD4 to CD8 ratio by flow cytometry was 3.9 to 1. The remainder of the patient’s hospital course was unremarkable. She was discharged home 5 days later with instructions to follow-up with her oncologist in the clinic.

After discussion with the patient and her family, she was started at our ambulatory oncology clinic on an R-CHOP (rituximab, cyclophosphamide, hydroxydaunorubicin, oncovin, and prednisone) regimen and intra-thecal methotrexate. A follow-up MRI after three cycles of chemotherapy showed an almost complete resolution of the paraspinal metastatic disease and epidural soft tissue extension (Fig. 1b). The patient continues to follow at our clinic 3 months after her diagnosis. She notes that her paresthesia had mostly disappeared. At time of writing, she is being prepared to undergo radiation therapy of the thoracic spine.

## Discussion

Extranodal lymphomas constitute 10-20% of all lymphomas [1]. Spinal lymphoma is a relatively uncommon spinal tumor and accounts for 1-2% of lymphomas’ extranodal occurrence [1] and 10% of epidural tumors [2]. Spinal lymphomas have been described in all age groups but are mostly a disease of the fifth to sixth decades of life [3]. It usually arises in the epidural or intramedullary spaces. The diagnosis of primary epidural spinal lymphoma requires the absence of any other identifiable site of lymphoma at the time of diagnosis [4]. Chronic back pain is the most common presenting symptom, with neurological signs appearing later in the disease course. B symptoms can also be present in a minority of patients [5].

In our case, malignancy was high on the differential diagnosis for a number of reasons. First, given the patient’s history of treated breast cancer, a metastatic spinal recurrence was initially highly suspected. Secondly, her history of Sjogren’s syndrome is a known significant risk factor for lymphoma. Third, treatment with adalimumab could also have precipitated the occurrence of lymphoma.

Non-Hodgkin’s lymphoma (NHL) accounts for 85% of spinal lymphoma cases, with the majority being diffuse large B cell lymphomas. These primary tumors are most commonly located in the thoracic spine, followed by the cervical spine and less commonly in the lumbar spine [6]. The predominance in the thoracic spine location is thought to be due to its plasticity, which is permissive for the development of bulky disease [7], in addition to its superior length and more concentrated lymphatic drainage [8]. Spinal lymphoma appears to be mostly a disease of males with a 1.6:1 male to female ratio [9]. Hodgkin’s lymphoma, T cell lymphoma, plasmablastic lymphoma and NK cell lymphoma have also been described in the spine [5]. Our described spinal lymphoma carries all classic characteristics related to age, sex, location, and pathological type.

The tumorigenesis of spinal lymphoma is not completely understood. It is thought to arise from the paraspinal soft tissues such as the paravertebral ganglions or the epidural lymphoid tissues [10]. It is then thought to enter the epidural space via the vertebral foramen without causing bony erosion [10]. This invasion method is likely to be unique to spinal lymphomas, in contrast to other tumors where cord invasion is achieved through vertebral bone destruction [11]. Rubinstein proposed that epidural lymphomas might arise from the epidural lymphoid rests through antigenic stimulation [12]. Unlike many lymphomas elsewhere, EBV does not seem to play a role in the development of spinal lymphoma [13].

When faced with a patient with a suspected lymphoma of the spine, it is first important to rule out other localizations of lymphoma with imaging, including positron emission tomography/computed tomography (PET/CT), in order to differentiate between primary spinal lymphoma and a secondary metastatic spread of a systemic lymphoma. The initial workup is similar to that of nodal lymphoma. Lack of bone destruction or erosion on CT or X-ray suggests the diagnosis of spinal lymphoma [3]. It is, however, not a specific or sensitive finding since bony erosion and destruction have been described [3]. The epidural localization of spinal lymphoma is more common and presents on MRI as iso- or hypo-intense homogenous tumor involving multiple segments with contrast enhancement and possible foraminal extension [14, 15]. The absence of hyper-intensity, although not a consistent finding, helps in differentiating it from metastasis or hematomas [14]. The yield of cerebrospinal fluid (CSF) analysis for cytology appears to be only modest even with repeat testing, and no particular CSF finding pattern is suggestive of spinal lymphoma [16]. For this reason, early biopsy is a reasonable option. Needle biopsy, even though extremely helpful for early diagnosis, unfortunately fails to achieve a satisfying diagnostic rate [5]. This fact is highlighted in our case where multiple CT-guided biopsies did not yield enough material or were inconclusive. The diagnosis was only established through surgical pathology. Tang et al proposed a diagnostic algorithm based on spinal instability: in the case of spinal instability or severe cord compression, primary surgical intervention with pathological examination is recommended. If the spine is deemed stable and in the presence of a mild to moderate compression, needle biopsy can be attempted and would be followed by a trial of chemo-radiation [5].

Treatment modalities lack robust evidence and involve chemotherapy, steroids, radiation therapy, stem cell transplant and surgery. As seen in our patient, a rapid neurological improvement following steroid administration is often seen, and is explained by lymphoma’s corticosteroid’s sensitivity. The literature offers conflicting evidence on the role of primary surgical decompression, in light of the tumor’s sensitivity to radiation and chemotherapy. For instance, Chang et al found that primary surgical decompression improved neurological outcomes in spinal cord compression due to diffuse large B cell lymphoma [17]. In contrast, Peng et al recommended a non-surgical management of cord compression related to spinal lymphoma, given higher post-operative mortality rates [18]. Another series did not show any benefits from surgery followed with radiation therapy as compared to radiation therapy alone [19]. Perry et al found that no patients were functionally worse after surgery [3]. In most series, the effect of surgery was confounded by the concomitant use of other treatment modalities such as chemotherapy and radiation therapy, which renders making a definite conclusion on the role of surgery complicated. Irrespective of the presence of cord compression, a surgical intervention is, as previously mentioned, often needed to establish a definite pathological diagnosis.

With regard to chemotherapy and radiation, the combination of both therapies is more effective than either of them alone. Hyper-CVAD (cyclophosphamide, vincristine, doxorubicin, and dexamethasone) or CHOP regimens have shown favorable outcomes [11]. The DeAngelis protocol which consists of pre-radiation high-dose methotrexate and post-radiation cytarabine has shown favorable outcomes [20]. Harris et al recommended an innovative algorithm in which primary neurosurgical intervention is pursued in the presence of severe neurological deficit or if tissue diagnosis is needed. In other cases, primary chemoradiation can be attempted, and surgical intervention can be performed in case of clinical deterioration [11]. Since our patient did not present with a disabling neurological deficit, conservative management was deemed appropriate. Surgery was eventually needed to establish a diagnosis and ensure spinal stability given the extensive nature of the disease. Adjuvant chemoradiation should still be considered after primary surgical intervention. A suggested regimen consists of six to eight cycles of CHOP or R-CHOP with radiation therapy at 40 - 55 Gy with 2 Gy per fraction [5].

Primary spinal lymphoma seems to have an unfavorable prognosis. The survival estimates that we found varied. In a series by Flanagan et al, the 2-year survival was found to be 36% [16]. Perry et al showed a more dramatic 1-year survival rate of 10% [3]. The median survival according to other sources is 6 - 9 months [3, 21]. Outcomes in immunocompromised patients are worse than their immunocompetent counterparts [22]. Poor prognostic factors include age more than 50, aggressive histological types, paraplegia, bladder and bowel involvement, poor performance status, elevated LDH and high protein in the CSF [23, 24].

Classically, central nervous system lymphomas were described in immunocompromised individuals. More cases are being described in immunocompetent patients, thus the need to include this entity in the differential diagnosis of any suspected spinal tumor in all hosts [25]. Clinicians should be aware that the diagnosis is usually delayed due to the similarity of presentation with other spinal diseases and the difficulty in obtaining a pathological diagnosis.

### Conclusion

We presented the case of an elderly female who presented with sub-acute back pain and was diagnosed with a primary spinal NHL. The diagnosis of this entity is complicated by the difficulty and delay in obtaining a satisfactory tissue sample. The treatment is incompletely defined due to the rarity of the disease. Physicians should keep this entity in the differential diagnosis of a newly diagnosed spinal tumor in order to hasten diagnosis and potentially improve outcomes.
